# Empyema Thoracis in a Patient Admitted with COVID-19: A Case Report

**DOI:** 10.31729/jnma.6473

**Published:** 2021-12-31

**Authors:** Angela Basnet, Sabin Chaulagain, Ashok Thapa, Manita Khadka, Manoj Khadka, Dhan Bahadur Shrestha

**Affiliations:** 1 Department of Internal Medicine, Scheer Memorial Adventist Hospital, Banepa, Kavrepalanchok, Nepal; 2 Nepalese Army Institute of Health Sciences, Sanobharyang, Kathmandu, Nepal; 3 Department of Internal Medicine, Mount Sinai Hospital, Chicago, IL, USA

**Keywords:** *COVID-19*, *empyema*, *pleural*

## Abstract

After almost a year of declaring COVID-19 a global pandemic, unusual presentations of the disease continue to be reported. Very little is known about its association with pleural disease. Here, we present a case of empyema thoracis in a 39-year-old male admitted with COVID-19. The pleural fluid later turned serosanguinous and eventually bleeding from other sites also occurred. During his treatment, antibiotics were given, thoracocentesis was performed and later thoracotomy was done. He died on the 19th day of admission following a hemorrhagic stroke. Pleural disease, although considered atypical and unusual presentation of COVID-19, needs careful and prompt diagnosis and earliest intervention. COVID-19, being a disease that involves multiple systems, and presentation of the disease may eventually lead to circulatory dysfunction and hence should be kept under consideration.

## INTRODUCTION

World Health Organization (WHO) declared COVID-19 a pandemic on March 11 2020^1^ since then, the disease has continued to unfold itself in severity. Nepal observed its first case on 13 Jan 2020.^2^ Although most people infected with the virus experience only mild to moderate respiratory illness and recover without specific treatment, many cases of complications and mortality have been reported.^3^ While much is yet to be studied about the disease, data regarding fatal thoracic empyema in COVID-19 patients is limited. Here, we present a case of thoracic empyema in a patient diagnosed with COVID-19 who later developed a hemorrhagic stroke.

## CASE REPORT

A 39-year-old man presented with high-grade fever, productive cough and shortness of breath on minimal exertion. There was no history of loss of taste or smell and no gastrointestinal symptoms. He was diagnosed case of hypertension and was under regular medication. He did not have any history of diabetes, tuberculosis or asthma. He consumed alcohol regularly but had no history of smoking.

On initial presentation, his vitals were significant for a fever of 100°F, blood pressure of 140/90mm Hg, respiratory rate of 22breaths/minute, pulse rate of 98beats/minute and oxygen saturation of 90% on room air. Systemic examination at the time of presentation was non-significant. Initial blood work revealed leukocytosis (total count of 11,000/mm^3^ with neutrophil predominance), Hemoglobin of 15.1gm/dl, platelets count of 150,000/cm and increased blood sugar level with fasting blood sugar of 162mg/dl and postprandial blood sugar of 212mg/dl. Given his presentation, he was subsequently tested with Reverse Transcription-Polymerase Chain Reaction (RT-PCR) for coronavirus which came back positive. He was then admitted to the isolation ward and was given remdesivir along with IV antibiotic ceftriaxone and regular insulin. Blood sugar was monitored. The oxygen saturation was maintained at 97% with 2-3 litres of oxygen via nasal prongs.

**Figure 1:**
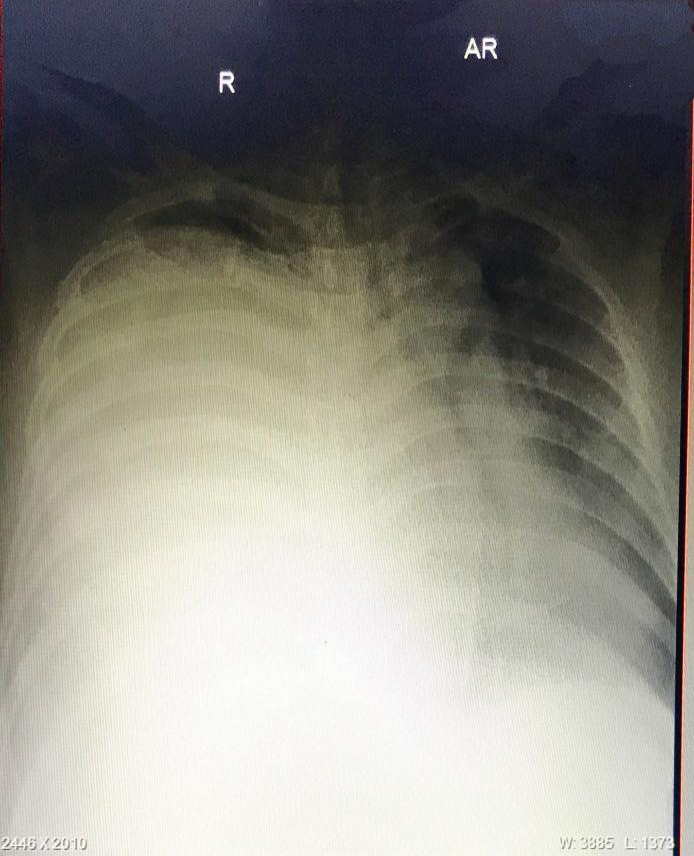
X-ray showing opacity of the right hemithorax.

On the 9th day of admission (11th December 2020), he had continuous fever with a maximum fever of 104°F and his oxygen saturation further decreased to around 70% without oxygen supplementation and maintaining to around 94% with oxygen at 8litres/min. Chest X-ray was significant for the opacity of the right hemithorax, suggestive of fluid (Figure 1).

**Figure 2:**
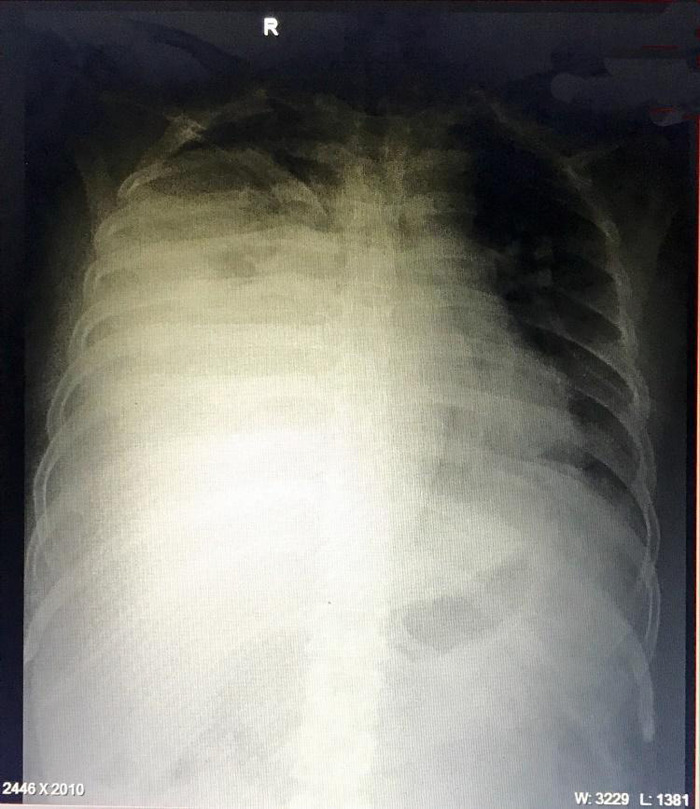
X-ray showing opacity of the right lung.

Subsequent CECT chest showed gross pleural effusion on the right side with mediastinal shift and displacement of the right hemidiaphragm. Diagnostic and therapeutic thoracocentesis was done with drainage of 2.7litres of thick, turbid orange-coloured fluid. Fluid analysis revealed leukocytosis with neutrophil predominance, the pleural fluid culture showed no growth after 72hours and LDH of 1858IU/l. Gene Xpert for tuberculosis and acid-fast bacilli in the fluid was negative. Following thoracocentesis, a chest X-ray was repeated which showed blunting of the right costophrenic angle but otherwise clear lung parenchyma. Antibiotics were changed to meropenem and metronidazole while continuing symptomatic treatment. On the 13th day of admission, his situation further deteriorated with an increased requirement for oxygen and continuous fever. Chest X-ray was repeated which showed opacity of the right lung field suggestive of pleural effusion (Figure 2).

Blood work revealed leukocytosis with a total count of 17,200/mm^3^. A chest tube was inserted and 2litres of orange turbid fluid was drained. Three days later, the drain tube was blocked and was noticed to contain serosanguinous fluid with blood clots.

The patient was referred to the cardiothoracic centre on 18th December 2020 for further management where the patient underwent thoracotomy. The bleeding from the chest tube could not be stopped during his stay there and he was found to have developed bleeding from other sites as well with the passage of black stool. On 21st December 2020, the patient was diagnosed to have developed a hemorrhagic stroke and passed away on the same day.

## DISCUSSION

The primary presentation of COVID-19 infection is like that of viral pneumonia or influenza-like illness, with about 20% of these presenting with severe or critical manifestation.^4^ COVID-19, being predominantly a disease of the respiratory system, results in an array of respiratory complications including severe pneumonia being the commonest and serious clinical manifestation. Around 20% of pneumonia cases develops parapneumonic effusion that may progress to empyema.^5,6^ Pleural disease is considered atypical and unusual presentation of COVID-19 but when present physicians are compelled to seek an alternative diagnosis.^7^ It was observed that about half of COVID-19 patients had comorbidities, therefore, there are arguments as to the pleural disease in COVID-19 could be related to the comorbidities rather than the virus itself.^8^ The empyema thoracis in our patient could have been precipitated by uncontrolled type 2 diabetes and an immunocompromised state due to chronic alcohol intake.

Empyema thoracis is defined as the collection of pus in the pleural space having multifactorial pathogenesis. It has been subdivided into three stages according to its course of evolution: Exudative, Fibrinopurulent and Chronic organizational stage.^9^ The pleural space in healthy adults contains a small amount of pleural fluid that acts as a lubricating film between two pleural surfaces. The pleural fluid accumulates when the rate of formation exceeds the rate of absorption when the permeability of capillaries is increased when the lymphatics are obstructed or due to decreased pleural pressure as in advanced empyema.^10^

Empyema needs to be treated early and aggressively from the time of diagnosis. Or else, complications may arise leading to sepsis, shock or death.^9^ The only reported case of thoracic empyema in COVID-19 patients explain the empyema to be secondary following aspiration due to the patient's history of achalasia.^11^ However, we cannot find a definite cause in our patient other than the comorbidities like diabetes and chronic alcohol intake.

Coagulation dysfunction seen in a higher proportion of COVID-19 patients is very well supported by evidence.^5^ Studies have shown that coagulopathy can be more prominent in critically ill cases.^12^ As shown by the case series from Italy, stroke, either ischemic or hemorrhagic, can develop in patients with severe pneumonia and multiorgan failure resulting in a poor outcome. As seen in our patient, when the equilibrium shift towards coagulopathy hemorrhagic stroke is likely along with other features of coagulopathy.^13^

This suggests that COVID-19 has wide involvement and can progress to life-threatening complications within a few days. The pleural disease though uncommon needs further study and the earliest aggressive intervention at the first suspicion of pleural effusion are recommended. The possibility of the patient going into coagulopathy and hemorrhagic stroke should also be considered.
